# Evidence that homozygous PTPRD gene microdeletion causes trigonocephaly, hearing loss, and intellectual disability

**DOI:** 10.1186/s13039-015-0149-0

**Published:** 2015-06-16

**Authors:** Nancy Choucair, Cecile Mignon-Ravix, Pierre Cacciagli, Joelle Abou Ghoch, Ali Fawaz, André Mégarbané, Laurent Villard, Eliane Chouery

**Affiliations:** Unité de Génétique Médicale et Laboratoire Associé INSERM à l’Unité UMR_S 910, Faculté de Médecine, Université Saint-Joseph, rue de Damas B.P. 17-5208 Mar Mikhael, Beyrouth, 11042020 Lebanon; Aix-Marseille Université, GMGF, Marseille, France; INSERM, UMR_S 910, Marseille, France; Département de Génétique Médicale, Assitance Publique Hôpitaux de Marseille, Hôpital d’Enfants de La Timone, Marseille, France; Neuropediatrics Department, Lebanese University, Beirut, Lebanon; Institut Jérôme Lejeune, Paris, France

**Keywords:** Protein tyrosine phosphatase receptor delta, Intellectual disability, Hearing loss, Trigonocephaly, Monosomy 9p

## Abstract

**Background:**

The premature fusion of metopic sutures results in the clinical phenotype of trigonocephaly. An association of this characteristic with the monosomy 9p syndrome is well established and the receptor-type protein tyrosine phosphatase gene (*PTPRD),* located in the 9p24.1p23 region and encoding a major component of the excitatory and inhibitory synaptic organization, is considered as a good candidate to be responsible for this form of craniosynostosis. Moreover PTPRD is known to recruit multiple postsynaptic partners such as IL1RAPL1 which gene alterations lead to non syndromic intellectual disability (ID).

**Results:**

We describe a 30 month old boy with severe intellectual disability, trigonocephaly and dysmorphic facial features such as a midface hypoplasia, a flat nose, a depressed nasal bridge, hypertelorism, a long philtrum and a drooping mouth.

Microarray chromosomal analysis revealed the presence of a homozygous deletion involving the *PTPRD* gene, located on chromosome 9p22.3. Reverse Transcription PCR (RT-PCR) amplifications all along the gene failed to amplify the patient's cDNA in fibroblasts, indicating the presence of two null *PTPRD* alleles.

Synaptic PTPRD interacts with IL1RAPL1 which defects have been associated with intellectual disability (ID) and autism spectrum disorder. The absence of the PTPRD transcript leads to a decrease in the expression of *IL1RAPL1.* These results suggest the direct involvement of PTPRD in ID, which is consistent with the PTPRD -/- mice phenotype.

Deletions of PTPRD have been previously suggested as a cause of trigonocephaly in patients with monosomy 9p and genome-wide association study suggested variations in PTPRD are associated with hearing loss.

**Conclusions:**

The deletion identified in the reported patient supports previous hypotheses on its function in ID and hearing loss. However, its involvement in the occurrence of metopic synostosis is still to be discussed as more investigation of patients with the 9p monosomy syndrome is required.

## Background

Craniosynostosis is a developmental anomaly characterized by a premature closure of one or several cranial sutures, leading to alterations in skull shape. Its incidence is 1 in 2000 to 2500 births and can be either isolated or associated with different other features. Nonsyndromic craniosynostosis is a multifactorial condition caused by genetic and environmental contributions; whereas syndromic craniosynostosis is associated with one of more than 180 identified monogenic syndromes with known inheritance patterns and dysmorphic features [1–3].

Metopic suture synostosis, resulting in trigonocephaly, has an estimated incidence of 1:15,000 live births [1, 4]. Little is known about its etiology and different theories have been proposed. An association of trigonocephaly with chromosomal abnormalities, more specifically with monosomy 9p syndrome (OMIM 158170) is well established and the receptor-type protein tyrosine phosphatase gene (*PTPRD*), located in the 9p24.1p23 region, was considered as a good candidate to be responsible for this form of craniosynostosis [5, 6].

Here we present a male child with a homozygous microdeletion of the *PTPRD* gene. Our results support a direct involvement of this gene in intellectual disability (ID), hearing loss, and trigonocephaly.

## Case presentation

### Ethical statement

This study was carried out with protocols approved by the Institutional Review Board (IRB) on human experimentation at the Saint Joseph University.

## Case report

The patient is a 30 month old boy, the second child of healthy consanguineous Lebanese parents. He was born at term via Cesarean section after an uneventful pregnancy. At birth, he weighed 2000 g.

He was referred to our laboratory at the age of 19 months because of delay in developmental and language milestones. He couldn't bottom-shuffle nor utter meaningful words.

**Fig. 1:**
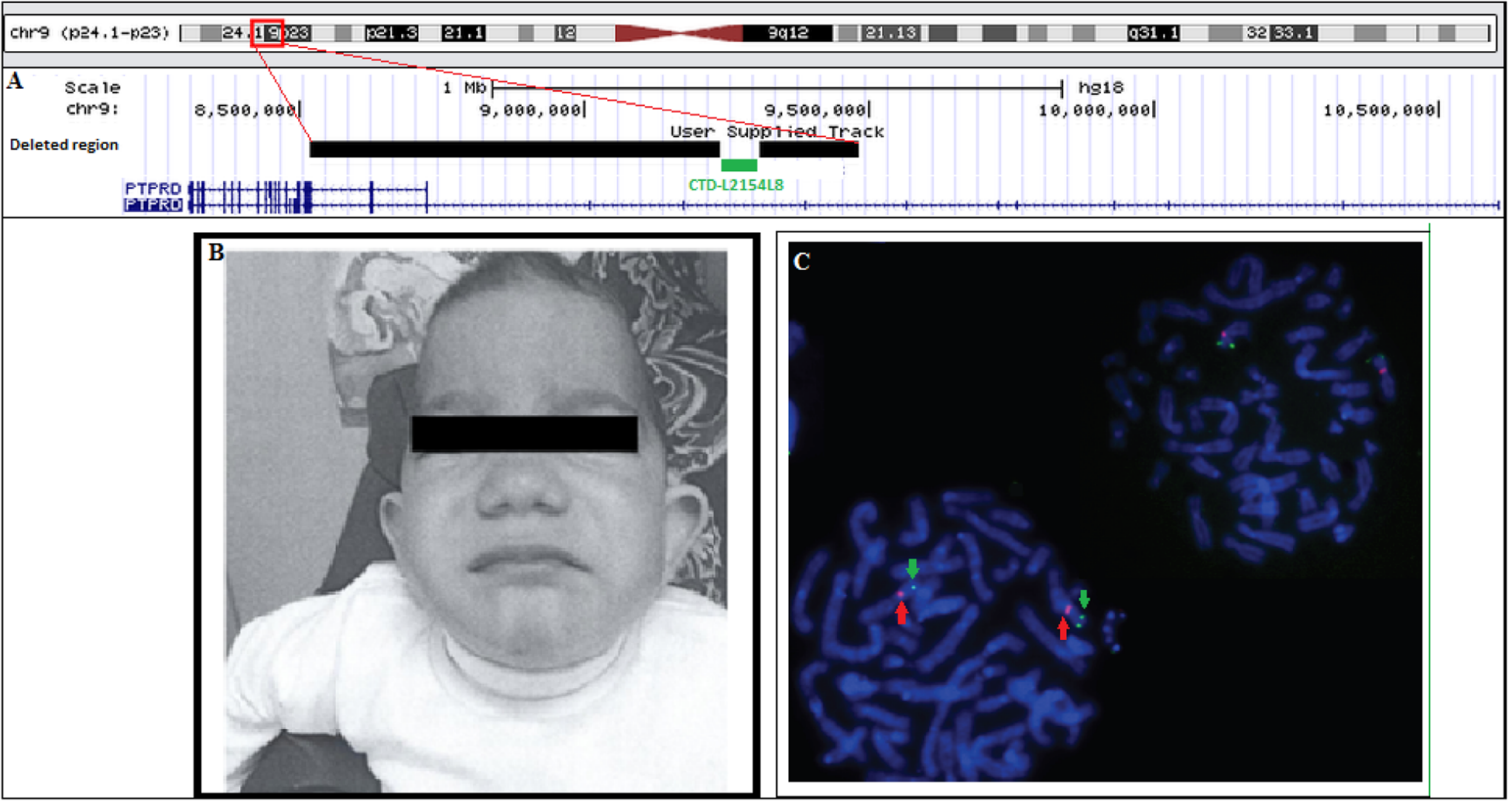
Clinical and genetic findings in the proband. **a** Representation of deleted regions within PTPRD. Black bars represent the deleted segments whereas the green one represents the BAC clone CTD-L2154L8 used in FISH. **b** Photograph of the proband at 3 years 6 months. The patient shows trigonocephaly, scaphocephaly, midface hypoplasia, a flat nose, a depressed nasal bridge, downslanting palpebral fissures, low set and large ears, a long philtrum, and a drooping mouth **c** FISH analysis confirming the presence of intron 8 on chromosome 9p. Arrowheads in red indicate a centromeric probe on chr9, and arrows in green indicate CTD-2154L8 mapping IVS8

He had a head circumference of 41.2 cm (<< 3rd percentile). Clinical examination showed microcephaly, trigonocephaly, scaphocephaly, midface hypoplasia, a flat nose, a depressed nasal bridge, downslanting palpebral fissures, hypertelorism, low set and large ears, a long philtrum, and a drooping mouth (Fig. 1b).

Brain magnetic resonance imaging (MRI) demonstrated a skull with a dolichocephalic pattern, a mild pachygyria of occipitoparietal lobes, and a mild widening of the frontal pericerebral subarachnoid space. The audiogram showed a bilateral hearing impairment with a hearing threshold at 40 dB.

At the age of 3 years and 6 months, his head circumference was 44 cm (<3rd percentile), his weight was 8000 g, and his height was 75 cm (< 3rd percentile).

## Results

Molecular karyotyping showed two adjacent deletions spanning the 9p24.1p23 region 46,XY.arr 9p24.1p23(8,527,597-9,248,069)x0,9p23(9,316,105-9,491,477)x0. Interestingly, these two interstitial homozygous deletions contained breakpoints within the *PTPRD* gene. They remove exon 9, a part of intron 9, and exons 10 to 15 of the gene (NM_002839) (Fig. 1a). No reported copy number variants that span these deletions were found in the Database of Genomic Variants (http://projects.tcag.ca/variation/).

**Fig. 2:**
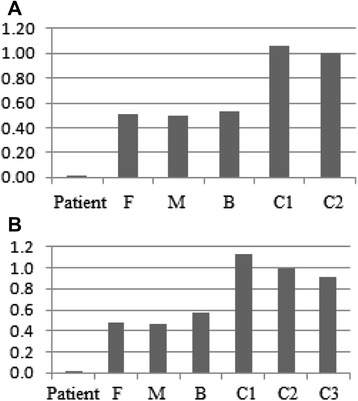
Dosage of *PTPRD* gene content using quantitative PCR. The bars represent the relative quantification of each sample. **a** The proband has zero copies of the first deletion within the PTPRD gene (chr9:8,527,597-9,248,069) and **b** zero copies of the second deletion touching this gene (chr9:9,316,105-9,491,477) whereas his parents and brother have a single copy compared to controls. P: patient, F: father, M: mother, B: brother, C1,C2, and C3: controls

Both deletions were confirmed using quantitative PCR and were found to be heterozygous in the proband's consanguineous parents and brother (Fig. 2). FISH analysis confirmed the presence of the undeleted intronic sequence of *PTPRD* on chromosome 9, ruling out a putative insertion or translocation of this sequence on another chromosome (Fig. 1c).

**Fig. 3:**
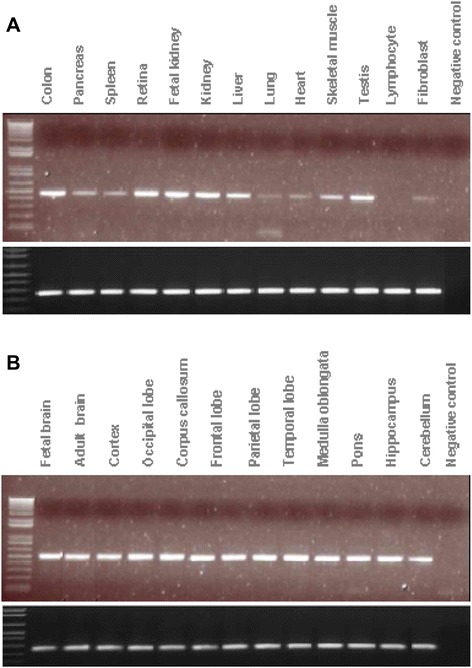
Expression of *PTPRD* in different tissues. Expression of *PTPRD* (top panels) and *GAPGH* (bottom panels) in **a** 13 human tissues and **b** 12 regions of the human brain. Several transcripts were found in the brain and in the heart, in addition to a high expression in the brain, testis, lung, retina, and colon. Conversely, PTPRD transcripts were found at low levels in the liver, kidney, and fibroblasts, and absent in lymphocytes

We next performed expression analysis in order to ascertain a potential causative role for PTPRD in the patient phenotype. We found that the PTPRD transcript is highly expressed in the brain. Several transcripts were found in brain and in the heart, in addition to a high expression in the testis, lung, retina, and colon. Conversely, PTPRD transcripts were found at low levels in the liver, kidney, and fibroblasts, and absent in lymphocytes (Fig. 3).

**Fig. 4:**
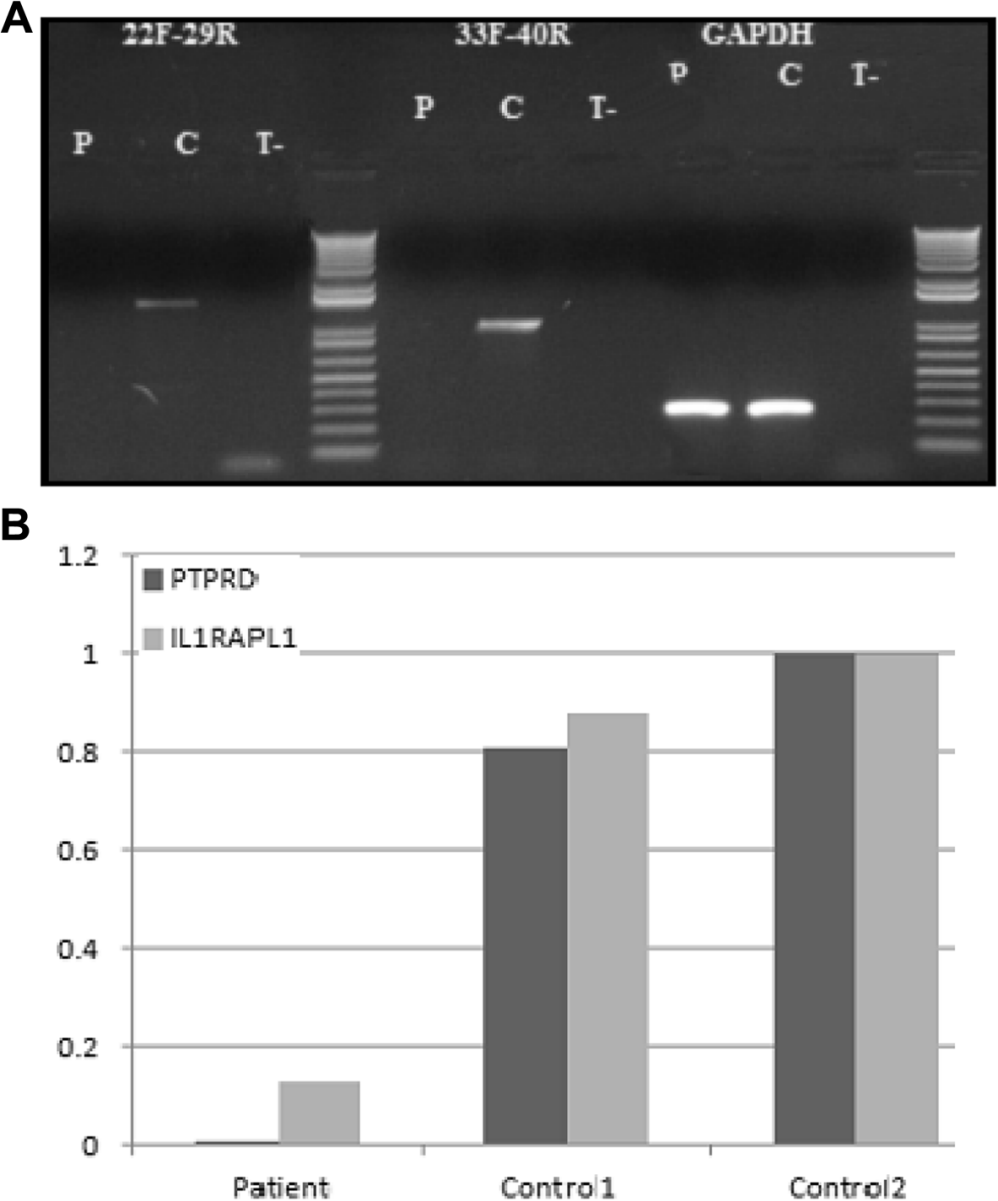
RT-PCR amplifications along the *PTPRD* gene and expression of *IL1RAPL1* in fibroblasts using Q-PCR. **a** RT-PCR amplifications indicate that no PTPRD transcript is found in the patient (P) in comparison to the control (C). *GAPDH* is used as an amplification control **b** Absence of the PTPRD transcript in the patient and a reduction of 92 % in the expression of IL1RAPL1 in fibroblasts

RT-PCR amplifications all along the *PTPRD* gene failed to amplify the patient's cDNA in fibroblasts, indicating that the deletions present in the patient are null alleles (Fig. 4a).

Finally, we studied the effect of the absence of PTPRD on the expression of IL1RAPL1 in fibroblasts because PTPRD and IL1RAPL1 interact and because mutations in the later are a cause of intellectual deficiency. We found that IL1RAPL1 transcripts are reduced by 92 % in the patient fibroblasts with respect to controls (Fig. 4b).

## Discussion

This study provides the first description of a *PTPRD* homozygous deletion, found in a patient with ID, growth retardation, hearing loss, trigonocephaly and scaphocephaly.

PTPRD belongs to the protein tyrosine phosphatase family, dephosphorylating phosphotyrosine, and playing essential roles in the regulation of receptor tyrosine kinase, growth, cell migration, and angiogenesis [7]. In particular, PTPRD plays an important role in excitatory and inhibitory synaptic organization. It is highly expressed in the brain and in scattered cortical neurons, presumptive interneurons, and CA2 hippocampal pyramidal cells [8].

Mouse and human PTPRD are 90 % and 96 % identical in the amino acid sequence of their extracellular and cytoplasmic regions respectively [9]. Knockout PTPRD mice exhibit growth retardation, posture and motor defects, early mortality due to reduced food intake, impaired memory and learning of spatial tasks, and enhanced long-term potentiation at hippocampal synapses, supporting the involvement of the PTPRD protein in synaptic functions [10, 11].

The complete absence of the PTPRD transcript in the patient described here might affect the postsynaptic recruitment of multiple postsynaptic partners such as IL1RAPL1. In mice, these protein-protein interactions regulate the excitatory and inhibitory synapses development [12].

Mutations in IL1RAPL1 cause non-syndromic X-linked ID. A neuronal overexpression of IL1RAPL1, through the trans-synaptic indispensable interaction with PTPRD, not only enhances excitatory presynaptic inputs but also increases dendritic spine formation and clustering of excitatory postsynaptic proteins. This synaptogenic activity is abrogated in cortical neurons from PTPRD-deficient mice [12, 13].

The study of the effects of this deletion on the synaptic PTPRD-IL1RAPL1 interaction in the patient was not possible. However, we found a reduction of 92 % of *IL1RAPL1* in the patient's fibroblasts tissue, supporting an involvement of the loss of PTPRD in the patient's ID.

Hemizygous deletions involving PTPRD were described in probands with ADHD or autism. Both cognitive disorders are acknowledged to be a common comorbidity for individuals with intellectual disability [14–16].

On the basis of these data, PTPRD was considered as responsible for the ID observed in the patient. However, its direct involvement in craniosynostosis has never been reported to date.

**Fig. 5:**
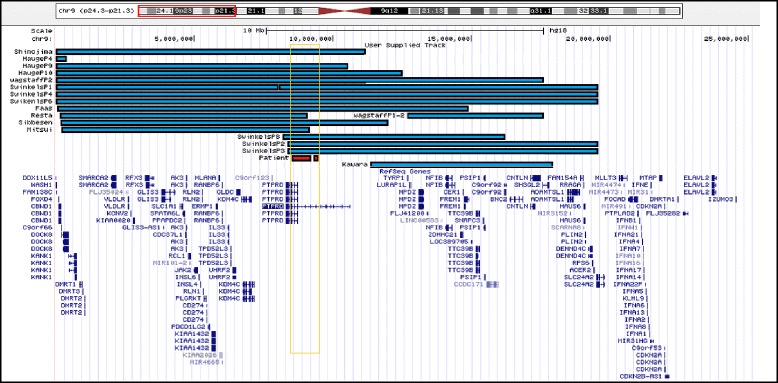
Graphical mapping of molecularly 9p24 interstitial deletions diagnosed in previously reported patients with trigonocephaly (blue bars) and in the patient here described (red bar)

Studies on patients with monosomy 9p proposed *PTPRD* as a candidate gene for metopic synostosis (OMIM 158170). Affected individuals have moderate to severe ID, developmental and psychomotor delay, midface hypoplasia, long philtrum, and trigonocephaly [17]. Several authors reduced the minimal critical region responsible for this syndrome to the *PTPRD* gene (Fig. 5) [6, 18].

The patient described here shows similar clinical features to those with monosomy 9p, especially trigonocephaly and ID. However, the existence of heterozygous deletions in the healthy consanguineous parents, brother, and in some 9p patients showing no trigonocephaly, suggests two hypotheses. On one hand, a possible recessive autosomic transmission mode for this characteristic, which imposes the contribution of additional mutations of *PTPRD* or regulatory elements or genes in the occurrence of trigonocephaly in the patient here described and other probands; especially those with monosomy 9p previously reported. On the other hand, this form of metopic synostosis is frequently unrecognized or misdiagnosed during infancy of which possibly the 9p monosomy patients or patient's family, as metopic ridging without synostosis is very common and is estimated to occur in 10–25 % of normal infants and young children [19].

In addition, some members of the PTPRD family of proteins are considered as regulators of craniofacial morphogenesis. *PTPRE* is an example, recognized to be implicated in the formation and differentiation of osteoblasts and whose haploinsufficiency increases trabecular bone mass due to cell-specific defects in osteoclast function in young females [20]. As some family members may retain similar function, further studies are recommended to confirm the involvement of PTPRD in trigonocephaly.

Loss of PTPRD expression could also be responsible for the patient's hearing loss. Indeed, a genome-wide association study in European populations suggested an association between hearing impairement and a variation in PTPRD (rs10815873; NM_002839.3:c.4086 + 398C.A) [21, 22]. *PTPRD* is expressed in the outer and inner hair cells, in the stria vascularis, the spiral ganglion, vestibular hair cells, and supporting cells, and was therefore considered a good candidate gene due to its expression pattern and the fact that it belongs to a family of proteins previously implicated in auditory function.

## Conclusions

The wide contribution of this gene in the phenotype of the patient supports many previous hypotheses on its function in ID, hearing loss, and trigonocephaly. However, its involvement in the occurrence of metopic synostosis is still to be discussed as more investigation of patients with the 9p monosomy syndrome is required. Meanwhile, parents were advised of a recurrence risk of 25 % of having another affected baby.

## Methods

### Cytogenetic and molecular techniques

#### Chromosomal microarray analysis (CMA)

Chromosomal microarray analysis was performed on genomic DNA isolated from peripheral blood lymphocytes (Kit Nucleospin Blood L, Macherey-Nagel, Eurl, Hoerdt, France) using an Affymetrix Cytogenetics Whole Genome 2.7M Array and following prescribed Affymetrix® protocols. Chip analyses were carried out using Affymetrix Chromosome Analysis Suite software (ChAS v.1.0.1) [23]. A Nimblegen array was also used in order to confirm the data generated (Roche Nimblegen, Madison, WI, U.S.A.). Genomic sequences were studied using the UCSC Genome Browser database (UCSC GRCH37/hg19).

#### Real-time quantitative PCRs (RQ-PCR)

CNVs were validated by RQ-PCR via the ΔΔCT method, using SYBR Green (Applied Biosystems, Life Technologies, Carlsbad, CA, USA) on an ABI 7500 real time PCR system. RQ-PCRs for the proband, his parents, his healthy brother, and several Lebanese reference samples were run in triplicate. Primers were designed using Primer Express 3 (ABI) for the quantification of *PTPRD*. α-Actinin2 *(ACTN2)* and adenosine receptor 2b (*ADORA2B*) were considered as endogenous genes. To exclude the presence of unspecific products, a melting-curve analysis of the products was performed after completion of the amplification.

### BAC DNA preparation and Fluorescence in situ hybridization (FISH)

Fluorescence in situ hybridization was carried out to confirm the presence of the undeleted intron 9 of *PTPRD* (NM_002839), as detected by the Cyto2.7M array. FISH was performed on fixed metaphase chromosomes obtained from the patient's peripheral blood culture. BAC clone DNA (CTD-2154L8) (provided by Life technology, France), selected using UCSC genome browser to be in intron 9 of PTPRD, was labeled with spectrum Green-dUTP, using a nick translation kit (Abbott Diagnostic, Rungis, France) according to the instructions of the manufacturers. A centromeric probe on chromosome 9 was labeled with spectrum Orange-dUTP. Hybridization was performed using standard procedures. Chromosomes were counterstained with 4,6-diamino-2-phenylindole (DAPI). Digital images were taken using an Axioplan-2 Zeiss fluorescent microscope (Zeiss) and a CCD camera (Photometrics « SenSys »).

#### Reverse transcription (RT-PCR)

The expression profile of *PTPRD* was studied by Reverse Transcription on two panels of total RNAs (BD Biosciences, Palo Alto, California, USA): 13 extracted from human tissues and 12 from human brain areas. Complementary DNAs (cDNA)s were synthesized using the SuperScript II Reverse Transcriptase (Invitrogen Life Technologies, Carlsbad, CA, USA) according to the manufacturer’s protocol. Primers were picked to highlight the presence of the different length transcripts of *PTPRD* obtained from UCSC Genomic Browser and designed through the Primer3 software.

#### RNA isolation and cDNA synthesis

The patient's fibroblast cell lines were grown and maintained in DMEM media under standard tissue culture. Total RNA was extracted from the patient’s cells using the PerfectPure™ RNA Purification System - 5 PRIME, and then transformed into cDNA as described above.

cDNAs of the patient and of a reference were amplified, using random primers from Eurofins MWG Operon, Germany, following these reaction cycling conditions: 94 °C for 2 min, 35 cycles of 94 °C for 30 sec, 60 °C for 30 sec, and 72 °C for 45 sec, with a post-cycling final extension of 8 min at 72 °C.

#### Real-time quantitative PCR on cDNA extracted from fibroblasts

The expression of IL1RAPL1*,* a protein that directly interacts with the Immunoglobuline like (Ig-like) domains of PTPRD, was studied. Specific primers were designed through the Primer Express 3.0.1 software as follows: IL1RAPL1-Forward: 5'-GCCAACCAGAGAACCTGAAATC-3' and IL1RAPL1-Reverse: 3'-CTTGGCCTCCATGTTTTTGTC-5', PTPRD-Forward: 5'-AAGGGAATTCAAGGTCACAGATG-3' and PTPRD-Reverse: 3' CAGTGAACTGGAACTGCCTTACTG-5'. The reaction cycling conditions were 95 °C for 10 min, followed by 40 cycles of: 95 °C for 15 sec and at 60 °C for 1 minute. Sequence Detection Software (SDS) was used for exporting and analyzing differences in Ct values (ΔCt) between the test locus and the control locus. c-abl oncogene 1, receptor tyrosine kinase *ABL1* was used as an endogene.

## Consent

Written informed consent was obtained from the patient for publication of this Case report and any accompanying images. A copy of the written consent is available for review by the Editor-in-Chief of this journal.

## Abbreviations

RT-PCR: Reverse Transcription PCR
PTPRD: Receptor type tyrosine protein phosphatase delta
ID: intellectual disability
MRI: Brain magnetic resonance imaging
CMA: Chromosomal microarray analysis
ChAS: Affymetrix Chromosome Analysis Suite software
RQ-PCR: Real-time quantitative PCRs
ABI: Applied Biosystems
ACTN2: α-Actinin2
ADORA2B: adenosine receptor 2b
FISH: Fluorescence in situ hybridization
IL1RAPL1: interleukin-1 receptor accessory protein-like 1 precursor
ADHD: Attention Deficit Hyperactivity Disorder
PTPRE: receptor-type tyrosine-protein phosphatase epsilon
IRB: Institutional Review Board
CNRS: Lebanese National Council for Scientific Research
